# Making the case for directed organ donation to registered donors in Israel

**DOI:** 10.1186/2045-4015-3-1

**Published:** 2014-01-23

**Authors:** Gil Siegal

**Affiliations:** 1 Center for Health Law, Bioethics and Health Policy, Kiryat Ono College, Kiryat Ono, Israel; 2 Gertner Institute for Epidemiology and Health Policy, Ramat Gan, Israel; 3 School of Law, University of Virginia, Virginia, USA

**Keywords:** Organ transplantation, Organ donation, Organ allocation, Israel, Ethics, Designated donation, Directed donation, Free riders, Priority, Optin/opt out

## Abstract

**Background:**

The number of deceased donor organ donations in Israel is lower than average when compared to other Western World countries. To address the organ gap, the 2008 Organ Transplantation Law provides new interventions, including important incentives to donors (and their families). The most notable of these was granting priority to registered donors (i.e., people on the waiting list who signed a donor card). The current study presents the normative arguments as well as the first documentation of public attitudes in Israel towards another possible incentive – allowing individuals to influence the allocation of their organs by permitting them to designate, to direct their donated organs [DD] to other registered donors, instead of the current allocation based primarily on medical criteria.

**Methods:**

A structured phone survey of 695 Israelis was conducted during Feb-March 2012. The sample is representative of the Israeli society in terms of age mix and gender, with adequate representation of the Arab and ultra-orthodox Jewish subgroups.

**Results:**

Among all Israelis, 68% stated a willingness to donate their organs, but only 16% reported to have already signed a donor card. 85% stated their interest in receiving an organ if the need arises. Overall, 64% of respondents felt that DD to a group of others who have registered as donors is justified, and the rate was remarkably higher in the Arab group (84%), and lower in the religious and ultraorthodox Jewish groups (52% and 50% respectively).

**Conclusions:**

The majority of the Israeli public supports organ donation and its proven benefits. Thus, organ recovery policy should be grounded in a strong communitarian strategy as we all stand to benefit from cooperation. However, current legislation and practices are modeled on individual disposition based on an opt-in legal framework. DD allows personal choices of to-be donors that might interfere with social interests, principles, and values such as equal access to care (i.e. organs) or justice. However, based on the result of this survey, the conceptual case of DD to other registered donors should be viewed more favorably (while the details should be addressed in future analyses), as DD is not expected to be rejected by the public at the outset. From a normative perspective, it is possible to support an allocation scheme that allows DD to other registered donors, where individual preferences that promote just sharing of the burden (donating organs) as well as the benefits (receiving an organ) of transplantation medicine are respected. Yet, DD to other registered donors should be understood and portrayed as a transition step towards a more communitarian model, and as a signal of solidarity by sharing organs as a public good rather than as an exercise of a quasi-property right.

## Background

Ever since the possibility of saving lives by using others’ organs became a reality, delineating acceptable avenues of retrieving these organs has been a matter of constant debate. The discussion becomes ever more intense due to an undeniable harsh truth – patients on waiting lists for organs die only because we cannot find them the vital organs they desperately need. Thus, the lethal unsupplied demand creates an ethical and social imperative to do better in meeting these patients’ needs [1]. The challenge is to design a policy that will increase the supply of organs without significant secondary harms (such as creating social segregation, increasing discrimination, or engaging in unethical conducts such as coerced retrieval).

Organs can be retrieved from live donors (in this case only non-vital organs such as one kidney, or partial liver or lung transplants), or from deceased donors, which obviates the physical harm to live donors but is associated with a wide range of psychological, religious, and behavioral obstacles to organ retrieval. This article pertains mostly to the latter option, and explores whether granting individuals some level of control over the disposition of their post-mortem organs (“directed, designated donation”, hereinafter DD) can increase the number of people willing to become donors, as family members rarely disregard the explicit wishes of donor card holders.

Several policies designed to increase organ donations have been implemented in recent years, including easier ways to document a willingness to donate (online registries, annexes to drivers’ license), massive public awareness campaigns, increasing live donation (mostly between family members), and offering various non-pecuniary benefits (as monetary incentives were rejected by most jurisdictions and international organizations) [1].

For many years, Israel has ranked lower-than-average in its retrieval rate from deceased donors; only 16% of adult Israelis in relevant ages signed donor cards and 45% of families consent to deceased donation, yielding a very low rate of 6-9 per million population (the current population is around 8 million), compared to 35, 25, 17 in Spain, France and the U.K. respectively [2]. As a result, Israel faces a chronic state of unsatisfied demand, with over 1,000 patients on the waiting list, some of them for over 4 years. Approximately 100 Israelis die annually only for the lack of a life-saving organ [3].

In 2008, the Israeli parliament (the Knesset) enacted a new Organ Transplantation Law designed to increase the performances and coordination of transplantation medicine in Israel, and to increase the availability of organs [4]. Among its provisions was an innovative plan for creating incentives for both live donation and deceased donation. Under the new law, *live* donors receive a uniform sum of money as compensation for the monetary loss reasonably attributable to procedures associated with organ removal and for the reimbursement of the donor’s expenses. In addition, donors are exempt from paying the health tax for a significant period of time and receive a Certificate of Recognition; they are also exempt from entrance charges to national parks.

As for *deceased* donors, the focus of this paper, the National Organ Transplantation Center bears their burial costs. The law also authorizes the Health Ministry to offer a reward to a person, or to his relative (either during the person’s lifetime or after his death) for agreeing to donate the deceased person’s organs. So far the Health Ministry has only authorized reimbursement of memorial expenses.

Importantly, the 2008 law authorizes the Steering Committee of the National Organ Transplantation Center to give priority to patients on the waiting list who have signed a donor card, should they or a first-degree relative need a transplant, as part of the effort to encourage the signing of donor cards and increase organ donations [5]. In other words, for the first time, the allocation criteria incorporate a non-medical criterion, a step which raises ethical concerns [6]. This policy has been in effect since April 2012, and during December 2012, a massive multilingual media campaign was launched to inform the public of their chance to receive preferred status on the waiting list by signing donor cards. The effect of ‘preferred status’ on the size of the donors’ pool remains to be studied, although early indications demonstrate a positive effect [7].

### Designated/directed donation

DD refers to the ability of a prospective donor to influence the allocation process by identifying designated recipient/s. DD can be complete/inclusive (where all organs are designated), whereas in *partial DD* the donor indicates her wishes regarding the disposition of a particular organ, leaving other organs to the general allocation pool. The underlying supposition is that some people refrain from donation because they do not know who will receive their organs. Reasons for wanting to designate vary – donating to a family member or a close friend in need, to a member of my race/religion etc. Clearly, it is easy to see how some of such reasons will generate resistance (e.g., racial/ethnic preference) while others seem [more] permissible (“first save my son”). Such people will be more willing to donate their (or their family member‘s) organs if the donor (or family members) has the prerogative to designate or influence who will be the recipient, as is currently the practice in live donation. Such a prerogative seems problematic, [8] especially in the unique Israeli context– a multi-ethnic, [9] multi-religious immigration society. Thus, carefully carving acceptable criteria for designation becomes essential [10].

We present the first documentation of public attitudes towards DD in Israel, where the criterion for designation is ‘DD to other Registered Donors’ [DDRD]. The designated group is open to all, thereby potentially negating claims about allocation based on discrimination, racism, social status etc. [11]. This is not the first probe of this option: In the US, the National Organ Procurement and Transplantation Network recognizes the legitimacy of DD, [12] and LifeShareres, an American nongovernmental organization, has been promoting DDRD, but with very limited acceptance – after 10 years, only some 16,000 individuals have joined this initiative [11,13]. In face of the inability to mobilize the American audience toward DD, it is of interest to evaluate the extent to which DD and DDRD resonate with the public in a different society.

One must differentiate ‘DD to other registered donors’ [DDRD] from ‘preferred status’ on the waiting list for registered donors. While the advantages given to registered donors seem similar, there are important differences between the two options. First, the locus of decision changes – while in ‘preferred status’ the allocating body accepts the donor’s organs and allocates them according to its criteria (which now include preferred status to organ card holders), DD allows the donor-to-be to shape the process and to exert some discretion on who gets her organs, an important demonstration of autonomy while she is alive (a will-like power). Second, ‘preferred status’ merely adds points to a candidate, and will be the decisive factor only if both candidates’ medical criteria are similar (same number of points by the allocation algorithm). In DD, someone lower on the waiting list but part of the “registered donors” club should get these organs first. While this might seem harsh at first sight, the corrective measure is simple – join the club (which is open to all). Finally, DDRD brings more forcefully to light the collective engagement needed to overcome the organ shortage –if we all prospectively join in, we all stand a much higher chance to get an organ if the need arises. Preferred status seems to reflect more of an individual risk management – the registration aims at improving individual’s chances over others’. DDRD attempts to minimize free riding on a larger, social scale by restricting non-donors’ access to organs.

## Methods and study design

**Figure 1:**
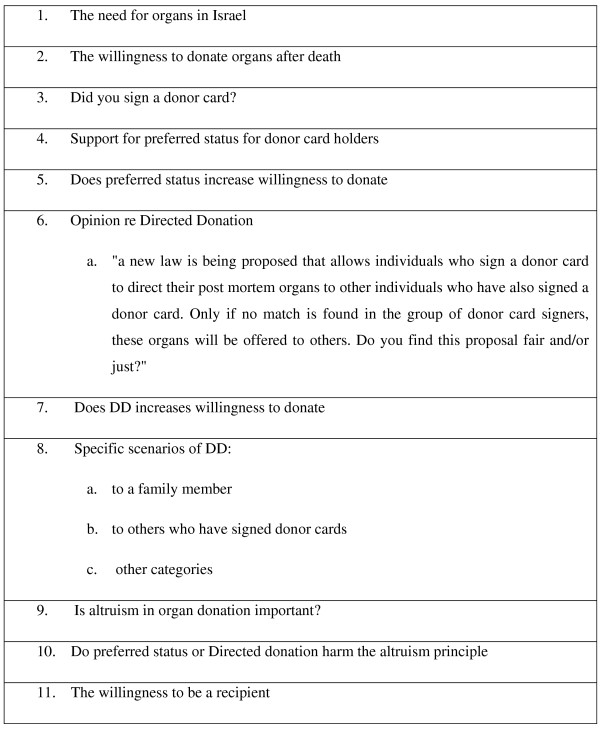
Main items surveyed.

(For detailed description of the methodology see Appendix 1 and Figure 1):

A structured phone survey of 695 Israelis containing 26 questions was conducted by Dahaf – Public Opinion Research Institute, Tel Aviv, during Feb-March 2012. The sample is representative of Israeli society in terms of age mix and gender (confidence level 95%, confidence interval 4%) with adequate representation of the Arabs and ultra-orthodox Jews subgroups. By ‘Israelis’ I refer to a legal status of individuals registered as being citizens of the State of Israel, being subject to its laws and being educated and cared for by Israel’s social institutions, including the healthcare system and its organ allocation scheme. The reader should bear in mind that Israel is a multiethnic and multi religious society. Many presumptions about “what do Arabs/Jews think about…” require empirical data, in order to focus educational attempt in a meaningful way, and not across the board, or to attune public campaigns to sensitivities of different subgroups.

The questionnaire data collection: Interviews were carried out during February - March 2012 using a computerized telephone system (CATI). Immigrants not fluent in Hebrew were interviewed in Russian, and Arabs were interviewed in Arabic. Each telephone number sampled was tried up to 3 times, at different days and hours. Only after 3 unanswered calls was another phone number substituted. Rate of success (finishing the entire survey) out of all telephone number sampled was 38%. To reduce the risk of bias and to maintain adequate sample representation, non-respondents were replaced by persons from the same sub-group/strata. There might still be some degree of bias if, within a strata, there is a correlation between the tendency to respond to phone surveys and attitudes toward organ donation.

## Results

In the following, the results presented reflect the entire Israeli public sample, unless a subgroup is specified.

### Awareness of the organ shortage

80 per cent of respondents were aware of the organ shortage in Israel – 83% among Jews, 64% among Arabs. Among Jews, the less religious were more aware of the shortage (religious and ultraorthodox Jewish 75 and 55% respectively).

### Willingness to donate, willingness to receive

Among all Israelis, 68 per cent stated they are willing to donate their organs – 66% among Jews (57% among former USSR immigrants and only 14% of Ultraorthodox Jews), and 77% among Arabs. However, only 16% said that they already had signed donor cards. The percentage of card holders in our study is comparable with the actual donor card holder rate in the National registry, which is a positive indication of our research sensitivity and representativeness.

Unsurprisingly, 85% of the entire sample stated their interest in receiving an organ if the need arises. It is particularly noteworthy that 65% of those who stated their refusal to donate were still interested in receiving organs.

### Attitudes towards priority status offered to registered donors

Among the entire sample, 55 per cent of the sample reported they have heard about the new law offering priority to registered donors. The comparable figure was only 38% among orthodox Jews, with no differences between former USSR immigrants or Arabs. Among the respondents *who had not yet signed* a donor card (84%), an important target for mobilization efforts, 44% stated that the law increased their willingness to donate, while 31% stated that it did not have this effect. The increase is noted primarily among those who did not sign a donor card but who have stated earlier that they are willing to donate their organ, and not among those who are unwilling to commit to donation (modest effect).

### Attitudes towards designated donation to other registered donors

64 per cent of the sample felt that DD to others registered as donors is justified (see Table 1). Remarkably, the support was significantly higher in the Arab group (84%), and lower in the religious and ultraorthodox Jewish groups (52% and 50% respectively).

**Table 1:** **Attitudes towards designated donation to other registered donors** [**DDRD**] (%)

	**All**	**Arabs**	**Secular Jews**	**Religious Jews**	**Ultraorthodox Jews**
Highly justified	22	25	25	18	13
Justified	42	59	40	34	37
Not justified	18	9	19	22	19
Highly unjustified	11	1	10	21	14
No answer	7	6	6	5	17
Total (%)	100	100	100	100	100

In respect to partial DD to a *family member* in need, 81 per cent found it justified (92% of Arabs, 72% Ultraorthodox Jewish respondents).

Assessing the impact of DD on the willingness of our sampled participants to sign a donor card among respondents who have not done so thus far, 52% stated it will significantly increase their willingness to sign (27% greatly increase, 25% increase). Importantly, another 28% stated that DD will not increase nor decrease their willingness to sign a donor card (thus DDRD does not seem to create a backlash). Breaking down the impact of the possibility to designate organs per group, DDRD had a positive effect on 44% of the Jewish population (20% greatly increase, 24% increase); 80% of the Arab population (51% greatly increase, 29% increase); and 44% in the Former USSR group (11% greatly increase, 33% increase).

Reasons for objecting to DD (29% of the entire sample) – 38% of those objecting DD stated that allocation should be based solely on medical needs, 25% felt that potential donors who would donate without signing a card should not be disadvantaged, 27% stated it is unfair/unethical (without specifications), and 1% stated that the willingness to donate should remain without consideration.

## Discussion

DD allows the prospective donor to exert some degree of control over his organs [14]. The practical implications of such an authority was evaluated by our survey. The results demonstrate that abiding to donors’ wishes can augment the number of potential donors, most notably in the Arab population of individuals who have thus far refrained for registering their consent to donate (80%). Thus, from a utilitarian perspective it is warranted.

However, a policy must also meet other requirements, including practicality, political feasibility and ethical scrutiny. In this article I shall consider the ethical issues, leaving the issues of practicality and political feasibility to others. This being said, the survey results play at least two important roles: they strengthen the case for DDRD by showing that it would be likely to increase organ donation rates. Our findings are particularly important in light of the discouraging US experience with Lifeshares. One might speculate that the lack of endorsement by the government plays a chilling effect on the willingness of individuals to join a private initiative in a delicate matter such as post mortem organs. They also increase our confidence in the political feasibility of the proposed change, as they demonstrate public support for it.

Ethical objection to DD rests on the following arguments: First, organs should be allocated based solely on objective medical criteria, which in turn reflects a perception of an altruistic donation and allocation system where the donor is permitting social agents (allocation committees) to administer just distribution of a scarce collective good. DDRD introduces a motivating factor (which restrict access to those who refuse or fail to participate in the consenting pool), which could be regarded as harming altruism, a notion strongly reigning in the organ donation discourse [15]. Second, the direct result of allowing DD is a discriminatory system, as it gives some individuals or groups (based on the type of designation at work) better access to a limited resource [16]. The downstream effect could be social segregation or even alienation, if patterns of cooperation or shirking would be grouped-based.

Such claims raise serious issues and require due attention. However, I would argue that most of them are not applicable under a well-intended, well-structured DD scheme, such as DDRD – directing my organs to peers who have chosen to undertake (if and when it becomes relevant) the same act of donation. First, Western bioethics adheres strongly to individual autonomy. Allowing individuals to designate their estate (use my money for X or Y) or to some extent their organs (to other registered donors) seems to be an act that extends individual autonomy in yet another regard. Indeed, our insistence on ‘consent to donation’ as oppose to ‘routine removal’ of cadaveric organs [17,18] is closely aligned with such recognition. Arguing that organs should be allocated solely on some socially agreed-upon criteria is strongly promoting the idea that my organs are actually a collective good. If this is the case – our loyalty to individual consent (opt in) should be diminished altogether, allowing some external interest (saving life, avoiding suffering, and reducing expenses) to have their say. In addition, we allow *live* donors to designate their organs as part of their autonomy, and it is hard to justify a complete retraction from some sort of individual’s control over his post-mortem organs (some retraction is defensible, as the live donor is putting himself at physical risk).

Second, altruism should be an ideal guide in many policy-making. However, a more realistic stance should reflect the understanding of the power and limitations of altruism. Indeed, the current deficiency is a vivid testimony to the insufficient clout of a strict “altruism-driven” scheme. Moreover, at the outset, it is not clear that DD, and especially DDRD (‘DD to other registered donors’) is actually harming altruism. The ability to direct one’s organs does not constitute an overt consideration, and a remote “benefit” to a donor (fulfilling his designation), especially in the circumstances of partial DD, seems over-righteous. In addition, it is almost impossible to assess “altruism” of donors under current policies (e.g., do we really question live donation among family members even though ample evidence point to near-coercion states in some cases?). Moreover, altruism has a negative impact on free riders – if all stand to benefit, some (or many) need not carry any burden, even though they would have opted-in if the stakes were high enough (i.e. the risk of being ineligible to receive an organ). The important contribution of DDRD is to reinforce the idea of “reciprocal altruism”— a public recognition of reciprocal obligation and interdependency in the transplantation domain (“we can all have a much higher chance of getting an organ if we all remain in the consenting pool”) that has been obscured by decades of exclusive emphasis on altruism and the “gift of life” [1].

Finally, the risk of discrimination must be confronted. DD is limited and directed towards sustaining donation—the permitted designated recipient groups refers to a group that is open to all, irrespective of social status, wealth, race, religion and similarly frowned upon criteria. For that very reason, DDRD should not be applied to minors or non-competent adults— they would be included in the eligible recipient group, as they should not be disadvantaged due to the inactions/omission of their guardians. Our suggested criterion is positively related to donation (i.e. not a whim or mal-intended) and is truly fair, as all Israeli citizens enjoy a national health care (unlike the American case, where all can be donors but only those with adequate insurance stand to receive organs). Thus, DD of one’s organs to the group of people like her, who agreed to share their organs, seems to me less objectionable.

As stated earlier in the introduction, DD to other registered donors [DDRD] is different from ‘preferred status’ on the waiting list for registered donors. While ‘preferred status’ only adds some points to a candidate, and will be the decisive factor only if both candidates’ medical criteria are similar, DD gives priority to someone lower on the waiting list, but who is part of the “registered donors” club. The corrective measure is simple – join the consenting club which is open to all. In such a scheme, DDRD could minimize free riding (accepting without willing to donate) by restricting access to organs to non-participants. In our study, 44% stated that preferred status increases their willingness to donate, and 64% felt that way about DDRD. It seems safe to assume that without the existence of ‘preferred status’ (which already advantages to some extent prospective donors), the effect of introducing DD could have been even higher.

Legal scrutiny of DD through the prism of current laws of the State of Israel is beyond the scope of this article and was presented in our recent project [19]. Suffice is to say that the gamut of laws pertaining to organ donation, healthcare rights, patients rights, and the Basic Laws on human rights do not preclude DD. Designated donation is permitted in *live* donation, and the Transplantation Law of 2008 does not ban deceased DD.

## Conclusion

Ideally, organ recovery policy would be grounded in a strategy that heralds and enhances cooperation (presumed consent) with an opportunity to opt out [20]. However, we now have a model of individual disposition based on an opt-in legal framework. DD represents a fascinating case study with the potential for a clash between personal choices (and the right for self-determination in life and upon death) on the one hand, and on the other hand social interests and values such as equality, parity and justice in policy making [14]. This paper provides needed data and normative arguments to support an allocation scheme that allows DDRD, where individual preferences that promote just sharing of the burden of donating organs as well as the benefits of transplantation medicine are respected, and that is likely to produce a higher proportion of prospective donors.

While beneficial in the short run, this approach could be problematic in the long run if it reinforces the existing moral and legal autonomy-driven model. If the DD approach were adopted, it should be understood and portrayed as a transition step toward a more communitarian model, and as a signal of solidarity of sharing of the organs as a public good rather than as an exercise of a quasi-property right (i.e., a limited right to decide over organ donation without a right to generate other gains), similar to the right given to the family to authorize or decline organ donation [1]. Indeed, the wished-for designated group should be as large as possible – hopefully the entire population. To this end, the legislator should opt for a legal rule that would make most people participate in the consenting pool. Identifying such a target points to the presumed consent/opt out default [1-18]. Finally, caution should be employed when attempting to generalize the results of the Israeli case to other societies. While the ethical analysis may stand ethical scrutiny, demonstration surveys in other countries are needed to assess the social support in each society.

## Appendix 1

### Research populations and samples

The population was defined as “all Israeli adult (age 18+) who are citizens”. Age distribution is represented in Table 2. From this population a representative sample of 700 interviewees was drawn. The sample was drawn using strata sampling method. Strata was defined by the following criteria: 1) Sector - Immigrants from the former Soviet Union that arrived in Israel since 1990 (hereafter cited as “immigrants”), Ultra orthodox Jews, Jewish residents in the West Bank (Settlers), Kibbutz members, other Jews, Arabs); 2) Characteristics of town of residence (Geographical area and size of town, according the categorization of Israeli CBS); 3) Gender. Sample size defined by Israel population (8 million), confidence level 95%, confidence interval 4%.

**Table 2:** Age distribution

**Percentage of respondents**	**Age group**
9.9	18-21
8.2	22-25
11.0	26-30
21.8	31-40
16.0	41-50
14.4	51-60
6.6	61-65
12.2	66+
100%	Total

Sampling immigrants applied the following additional criteria: 1) Republic of origin (Christian Vs. Islamic republics); 2) Year of immigration. Sampling in the Arab sector applied additional criteria: 1) Profile of town of residence; 2) religion: Moslem, Christian and Druze. From each stratum a random sample was drawn. Each stratum was presented in the sample according to its proportion in the population.

### The questionnaires

The structured questionnaire contained 26 questions. It was translated to Russian and Arabic, and checked by back translation from Russian/Arabic to Hebrew. The original translation and the back translation were made by different persons.

### Data collection

Interviews were carried out by during February - March 2012 using computerized telephone system (CATI). Immigrants not fluent in Hebrew were interviewed in Russian, Arabs were interviewed in Arabic. Each telephone number sampled was tried up to 3 times, at different days and hours. Only after 3 unanswered calls a substitute number was addressed. In cases of refusal, the “convincing efforts” adopted by Dahaf institute were administered. Interviewee who wanted to terminate the interview before completing the questionnaire was asked for his permission to address him later or the next day to complete the interview. Rate of success out of all telephone number sampled was 38%. To avoid bias and to maintain adequate sample representation, non-respondents were replaced by persons from the same sub-group/strata.

### Instructing the interviewers

All interviewers were face-to-face instructed in groups of up to 20 interviewers. Instruction was carried out in the following stages: Each interviewer read the questionnaire. One of the interviewers served as a “pretending interviewee” and was interviewed in front of the other interviewers. The pretending interviewer was instructed to give the real interviewer problematic responses (ask difficult questions, give contradicting answers, criticize the questionnaire, etc.). The instructor summarized all the problematic points and instructed the interviewers how to deal with them. The first 3 questionnaires of each interviewer were checked before proceeding with work. The check referred mainly to missing data or contradictions. Upon completion of data logical checks were carried out. During actual interviews, interviewers were monitored. An interviewer didn’t know when the supervisor was monitoring him. After the interviewer completed relevant questionnaire, the supervisor addressed him and presented corrections, if required.

### Controlling order-effect bias

A preliminary evaluation of possible order effect (were the sequence of items in a survey influences the responder’s attitudes) was carried by Dahaf’s statistical team. The pilot version was tested for such sequencing effect. To de-bias possible effect, identified questions were introduced in random sequence.

## Competing interests

The author declares that he has no competing interests.

## Author information

GIL SIEGAL, MD, LLB, SJD is a surgeon and a health law professor with the University of Virginia School of Law, Ono Academic College’s Center for Health Law and Bioethics, and the Gertner Institute for Health Policy and Epidemiology. Siegal is the Editor-in-Chief, Journal for Health Law & Bioethics (Heb). He received his medical and law degrees from Tel Aviv University and a Doctorate in Law from the University of Virginia.
